# A Sustainable and Efficient Synthesis of Benzyl Phosphonates Using PEG/KI Catalytic System

**DOI:** 10.3389/fchem.2016.00035

**Published:** 2016-08-16

**Authors:** Shamrao Disale, Sandip Kale, George Abraham, Sandeep Kahandal, Ashish N. Sawarkar, Manoj B. Gawande

**Affiliations:** ^1^Department of Chemistry, Institute of Chemical TechnologyMumbai, India; ^2^Department of Chemistry, SIES College of Arts, Science and CommerceMumbai, India; ^3^Department of Chemistry, B.N. Bandodkar College of ScienceMumbai, India; ^4^Department of Chemical Engineering, Motilal Nehru National Institute of TechnologyAllahabad, India; ^5^Department of Physical Chemistry, Faculty of Science, Regional Centre of Advanced Technologies and Materials, Palacky UniversityOlomouc, Czech Republic

**Keywords:** sustainability, KI +K_2_CO_3_, PEG, synthesis of benzyl phosphonates, green chemistry

## Abstract

An efficient and expedient protocol for the synthesis of benzyl phosphonates using KI/K_2_CO_3_ as a catalytic system and PEG-400 as benign solvent has been developed. The reaction proceeds smoothly at room temperature achieving excellent selectivity and yield of the corresponding products. The combination of PEG-400, KI, and K_2_CO_3_ in this reaction avoids the need of volatile/toxic organic solvents and reactive alkali metals or metal nanoparticles/hydrides. We believe that this benign combination (PEG-400 and KI) could be used for other related organic transformations.

## Introduction

Compounds containing C-P bonds are an attractive synthetic targets because of their significant applications as isosteric analogs of phosphate esters (Engel, 1977; Huang and Chen, 2000; Manabe and Kobayashi, 2000), target-specific modulators for biological processes (Engel, 1992), phosphonopeptides (Kafarski and Lejczak, 1991), amino acid analogs (Rushing and Hammer, 2001; Fredriksen and Amedjkouh, 2016), and as pro-drugs (Krise and Stella, 1996). Esters of phosphonic acids are key and important intermediates in a variety of synthetically important reactions such as Wadsworth-Emmons reactions (Quin, 2000), and they also act as chelating agents for many imperative metals (Anderson et al., 2002). Although phosphorus compounds containing the C-P bond are not particularly abundant in nature (Kittredge and Roberts, 1969), their formation still remains a formidable challenge.

Conventional methods for the synthesis of organophosphorus compounds are Michaelis-Arbuzov (Bhattacharya and Thyagarajan, 1981; Anna and Artur, 2015), and Michaelis-Becker reactions (Meisters and Swan, 1965). Michaelis-Arbuzov method involves the formation of a phosphonium intermediate through the nucleophilic addition of the phosphorus lone pair to the alkyl halide to form a new alkyl halide and the target product alkyl phosphonate. These conventional methods suffer from major drawbacks, when this newly formed alkyl halide is more reactive or less volatile than the initial alkyl halide used thus resulting in a mixture of phosphorylated products (Saady et al., 1995a,b). This reaction also requires high temperature, particularly for unreactive halides. In Michealis-Becker reaction, an alkali metal salt of dialkyl phosphate reacts with an alkyl halide under milder reaction conditions but requires strong bases (Kers et al., 1997). Sometimes a mixture of products are formed by single electron transfer (SET) mechanism due to the high reactivity of phosphate anion generated with the substrates having pseudo halide character (Michalski et al., 1991; Engel, 1992; Witt and Rachon, 1996). The reaction can also be carried out under phase transfer catalyzed conditions but the limitations of these methods are not clearly explained (Kem et al., 1981; Shi et al., 2000; Tomilov et al., 2001). Therefore, to alleviate these problems, other methods have been investigated (Lavén and Stawinski, 2009; Takahashi et al., 2009; Rajeshwaran et al., 2011; Bloomfield and Herzon, 2012; Xu et al., 2013; Wang et al., 2014) Hence, there is scope to develop new methodology or to improve the previous methods to form the P-C bond.

Polyethylene glycol (PEG) is an environmentally benign alternative reaction medium in synthetic chemistry (Chen et al., 2005), and used in various substitution, oxidation and reduction reactions. PEG and its monomethyl ethers have been widely used as phase transfer catalyst (PTC) in many organic reactions (Timko et al., 1977; Dickerson et al., 2002; Chandrasekhar et al., 2003), due to their unique advantages of high thermal stability, negligible vapor pressure, low cost, easy availability and recyclability. The high water miscibility of PEG facilitates its separation from reaction products when used as a reaction medium.

## Experimental methods

### Reaction procedure

To a stirred mixture of benzyl halide (1 mmol), dialkyl phosphite (1 mmol), K_2_CO_3_ (2 mmol), KI (0.3 mmol) and PEG-400 (0.5 g) was added. The reaction mixture was stirred at room temperature for 6 h. The progress of the reaction was monitored by TLC. After completion of reaction the product formed was then extracted with diethyl ether (2 × 10 mL). The obtained residual oil was further purified by using column chromatography (petroleum ether/ethyl acetate 10%).

## Results and discussion

**Scheme 1:**
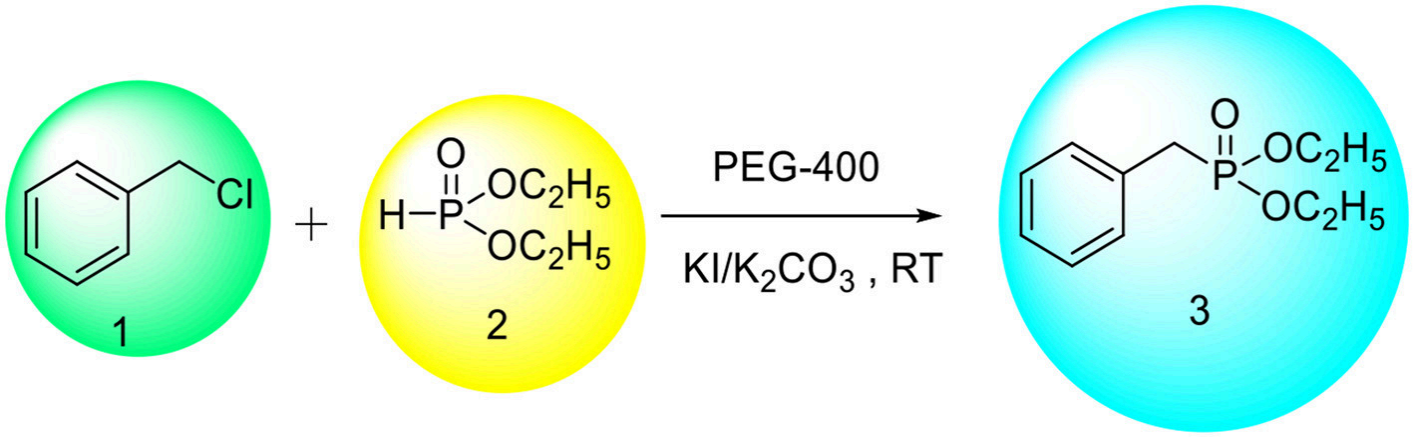
**Synthesis of benzyl phoshponates over PEG-400/KI.K_2_CO_3_ system**.

In continuation of our efforts for synthesis of organic compounds using sustainable protocols (Gawande et al., 2013a,b,c,d, 2014a,b,c; Kale et al., 2013; Sharma et al., 2015), in this work, we have successfully developed a mild and ecofriendly synthesis of benzyl phosphonates using PEG/KI as a catalytic system. In this protocol, PEG not only acts as a reaction medium but also as a PTC. We have used KI for *in situ* formation of benzyl iodide since it is inexpensive, nontoxic and readily available reagent. The use of KI avoids direct use of expensive benzyl iodide. Initially, we chose benzyl chloride (1 mmol) and diethyl phosphate (1 mmol) as model substrates to establish the optimum conditions for the reaction (Scheme 1).

We examined the effect of various solvents such as MeCN, DMF, THF, and PEG-400 at room temperature by adding K_2_CO_3_ as a base in the reaction. The reaction was found to be solvent dependent giving the best result in PEG-400 (Table 1, entry 12). PEG enhances the reactivity of the inorganic base by chelation of the countercation. To further improve the efficiency of this green synthetic approach, various carbonates and hydroxides of other alkali and alkaline earth metals were applied as bases to promote this transformation in PEG. Both cesium carbonate and KOH gave comparable yields of corresponding phosphonates along with benzyl alcohol as the side product and hence, we decided to use powdered anhydrous K_2_CO_3_. To improve the catalytic performance in terms of yield, KI was tested as an additive. In this case the addition of KI resulted in a significant improvement in the percent yield of the phosphonates. 0.3 eq. of KI and 2 eq. of K_2_CO_3_ were found to be the optimum amounts giving best results. The reaction conditions were then finally established (Table 1, Entry 12).

**Table 1:** **Effect of solvent and base on the reaction of benzyl chloride and diethyl phosphite^a^**.

**Entry**	**Solvent**	**Base**	**Yield^d^ (%)**
**EFFECT OF SOLVENTS**			
1	MeCN	K_2_CO_3_	23
2	DMF	K_2_CO_3_	45
3	THF	K_2_CO_3_	28
4^b^	–	K_2_CO_3_	20
5	PEG-400	K_2_CO_3_	60
**EFFECT OF BASES**			
6	PEG-400	Li_2_CO_3_	35
7^c^	PEG-400	Li_2_CO_3_	52
8	PEG-400	Na_2_CO_3_	48
9^c^	PEG-400	Na_2_CO_3_	61
10^c^	PEG-400	Cs_2_CO_3_	63
11	PEG-400	KOH	60
12^c^	PEG-400	K_2_CO_3_	97

a *Reaction Conditions - benzyl chloride (1 mmol) and diethyl phosphite (1 mmol), base (2 mmol), RT (28°C) 6 h*.

b *No solvent*.

c *Reaction with KI (0.3 mmol)*.

d *Isolated Yield*.

Once the optimum conditions have been established, a range of substituted benzyl halides (1 mmol) were reacted with various dialkyl phosphites (1 mmol) in the presence of KI (0.3 mmol) and anhydrous powdered K_2_CO_3_ (2 mmol) using PEG-400 as a reaction medium. The reaction mixture was stirred at room temperature for 6 h. Formation of the corresponding benzyl phosphonates occurs with good to excellent yields of the desired products. The versatility of this process was demonstrated with respect to various electron donating and electron withdrawing benzyl halides (Table 2).

**Table 2:** **Synthesis of substituted benzyl phosphonates in PEG-400^a^**.

**Entry**	**Benzyl halide**	**Product**	**Yield^b^ (%)**
1	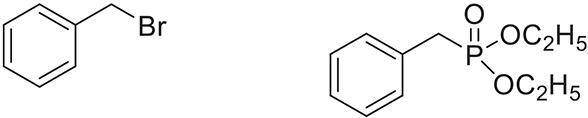		98^42^
2	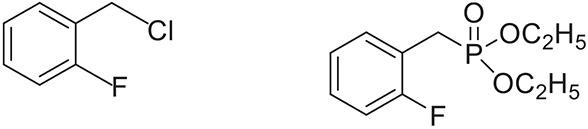		92^43^
3	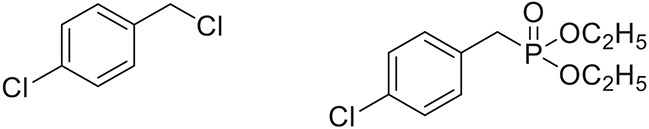		89^42^
4	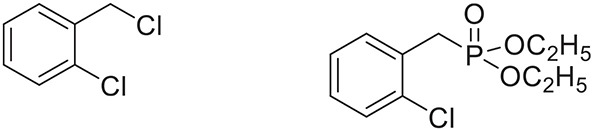		91^42^
5	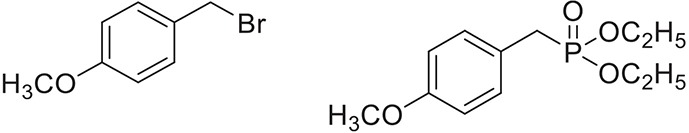		88^44^
6	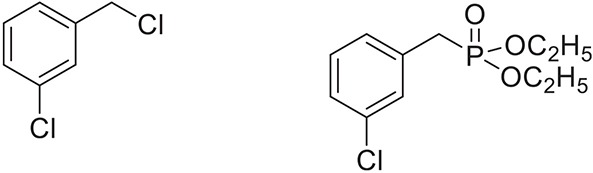		93^45^
7	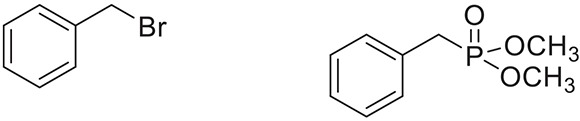		98^46^
8	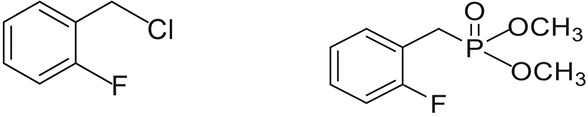		91^47^
9	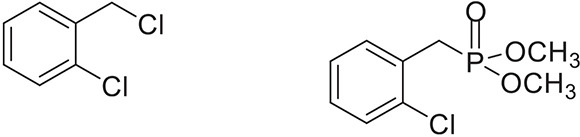		90^47^
10	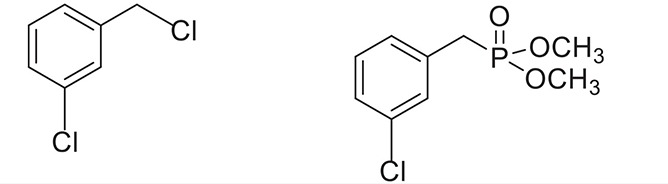		93^47^
11	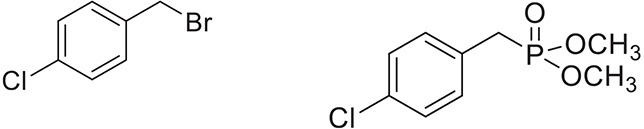		90^48^
12	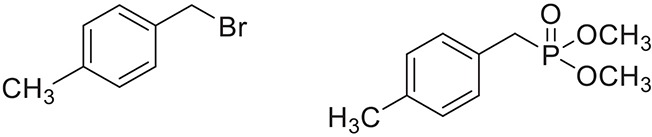		94^48^
13	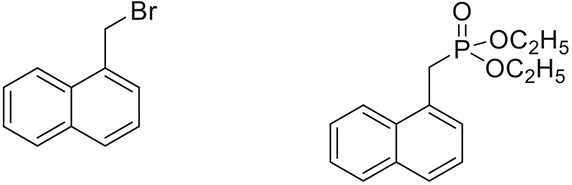		82^49^

a *Reaction Conditions: benzyl halide (1 mmol) and dialkyl phosphite (1 mmol), K_2_CO_3_ (2 mmol), KI (0.3 mmol), PEG (0.5 g), RT (28°C), 6 h*.

b *Isolated Yield*.

**Scheme 2:**
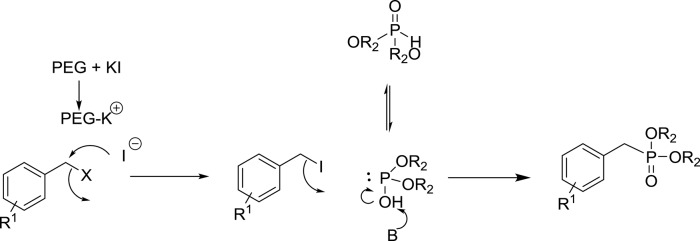
**Plausible mechanism for the formation of benzyl phosphonate**.

On the basis of experimental results and the literature, the possible mechanism for the formation of substituted benzyl dialkyl phosphonate is likely to take place in two steps (Scheme 2). The initial step, formation of benzyl iodide is likely to occur by Fenkelstain reaction, in which the chloride or bromide ion is replaced by the iodide. The PEG-400 may be facilitating this step by assisting the dissociation of KI. The possible role of PEG is to activate the anion by forming a complex with the cation similar to crown ether. This means that PEG enhances the nucleophilicity of the iodide ions and facilitates the conversion of benzyl chloride/bromide into benzyl iodide. To check the formation of benzyl iodide in the course of the reaction, we carried out the reaction between KI and benzyl chloride in the absence of K_2_CO_3_ and phosphite in PEG-400 and observed that benzyl iodide is formed in good yield which was further confirmed by GC and GCMS analysis (see Supplementary Material). In the second step of the reaction, the nucleophilic displacement of iodide takes place by the dialkyl phosphite to give the corresponding phosphonate.

In conclusion, we have developed a simple, efficient and sustainable protocol for the synthesis of benzyl phosphonates from benzyl halides using KI/K_2_CO_3_ in PEG-400. PEG-400 may be used as solvent for the Fenkelstein reaction instead of volatile acetone. The advantages include broad application scope, mild reaction conditions, excellent yields, simple operation, cost effectiveness and environmental friendliness which make this protocol a value addition to the existing methods in the synthesis of benzyl phosphonates.

## Author contributions

SD, SKal, and SKah worked on the experimental parts, GA, AS helped writing and MG is the supervisor of this research work.

### Conflict of interest statement

The authors declare that the research was conducted in the absence of any commercial or financial relationships that could be construed as a potential conflict of interest.

## Graphical abstract

An efficient and convenient method for the synthesis of benzyl phosphonates using KI/K_2_CO_3_ as a catalyst and PEG-400 as an environmentally benign solvent has been developed.
