# Supplementary material for: A Sustainable and Efficient Synthesis of Benzyl Phosphonates Using PEG/KI Catalytic System
Source: Front Chem. 2016 Aug 16;4:35. doi: 10.3389/fchem.2016.00035 (PMC4986413; doi:10.3389/fchem.2016.00035)

**Supporting Information**

**A sustainable and efficient synthesis of benzyl phosphonates using PEG/KI catalytic system**

Shamrao Disale,^a^ Sandip Kale,^b^ George Abraham,^b^  Sandeep Kahandal,^c^ Ashish N. Sawarkar,^d^ and Manoj B. Gawande^e^*

^a^Department of Chemistry, Institute of Chemical Technology, Nathalal Parekh Marg, Matunga, Mumbai, India. ^b^Department of Chemistry, SIES College of Arts, Science and Commerce, Sion, Mumbai, India.

^c^Department of Chemistry, B. N. Bandodkar college of Science , Thane, Mumbai, India.

^d^Department of Chemical Engineering, Motilal Nehru National Institute of Technology, Allahabad, Uttar Pradesh, India.

^e^Regional Centre of Advanced Technologies and Materials, Department of Physical Chemistry, Faculty of Science, Palacky University, Šlechtitelů 27, 783 71, Olomouc, Czech Republic.

Email [– mbgawande@yahoo.co.in](mailto:– mbgawande@yahoo.co.in) and [manoj.gawande@upol.cz](mailto:manoj.gawande@upol.cz)

**General Experimental procedure:**

To a stirred mixture of benzyl halide (1.0 mmol), dialkyl phosphite (1.0 mmol), KI (3 mmol), K_2_CO_3_ (2 mmol) and PEG-400 ( 0.5 g) was added. The reaction mixture was stirred at room temeparture for 6 h.The progress of the reaction was checked by TLC. After 6 h, the reaction mixture was poured in water. The product formed was then extracted with diethyl ether (2X10 ml) product. The residue oil obtained after concentrating the combined ether extract on rotary evaporator was further purifed by using coloumn chromatograhy (petroleum ether/ethyl acetate 10%).

**^1^H NMR Spectra of the Dimethyl benzylphosphonate:**

**
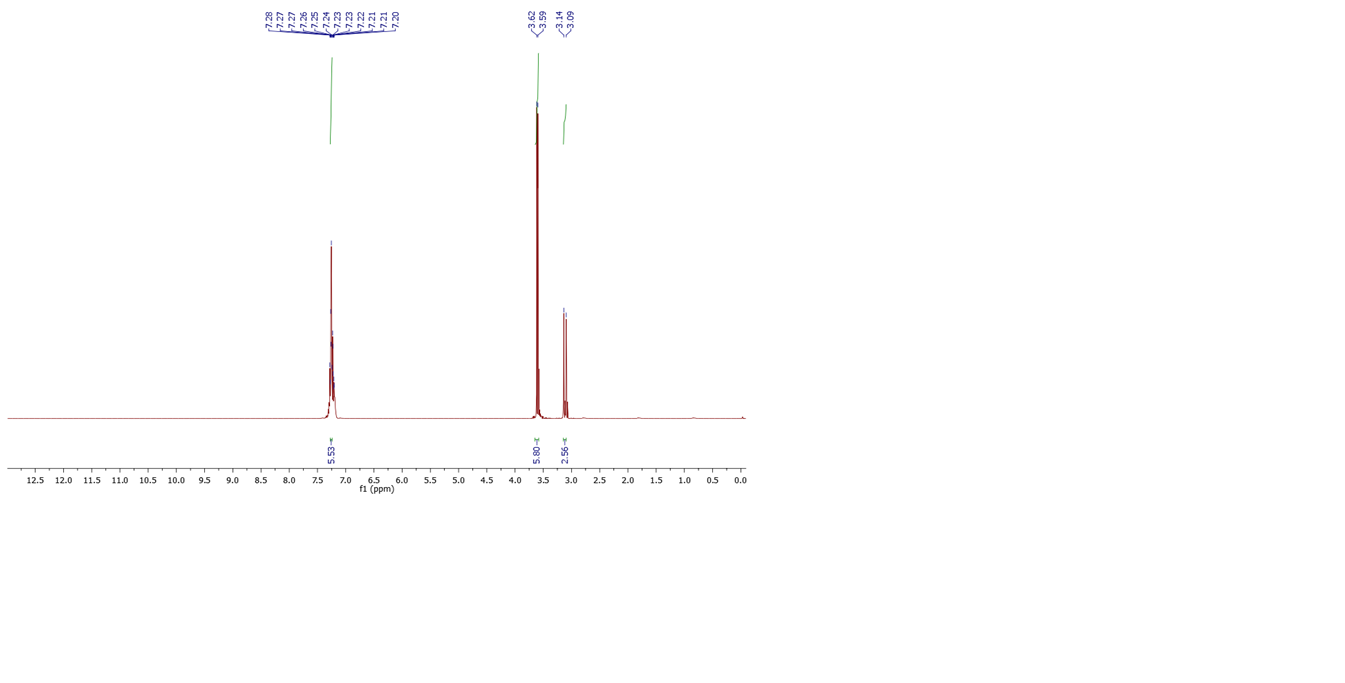
**

**^13^C NMR Spectra of the Dimethyl benzylphosphonate:**

**
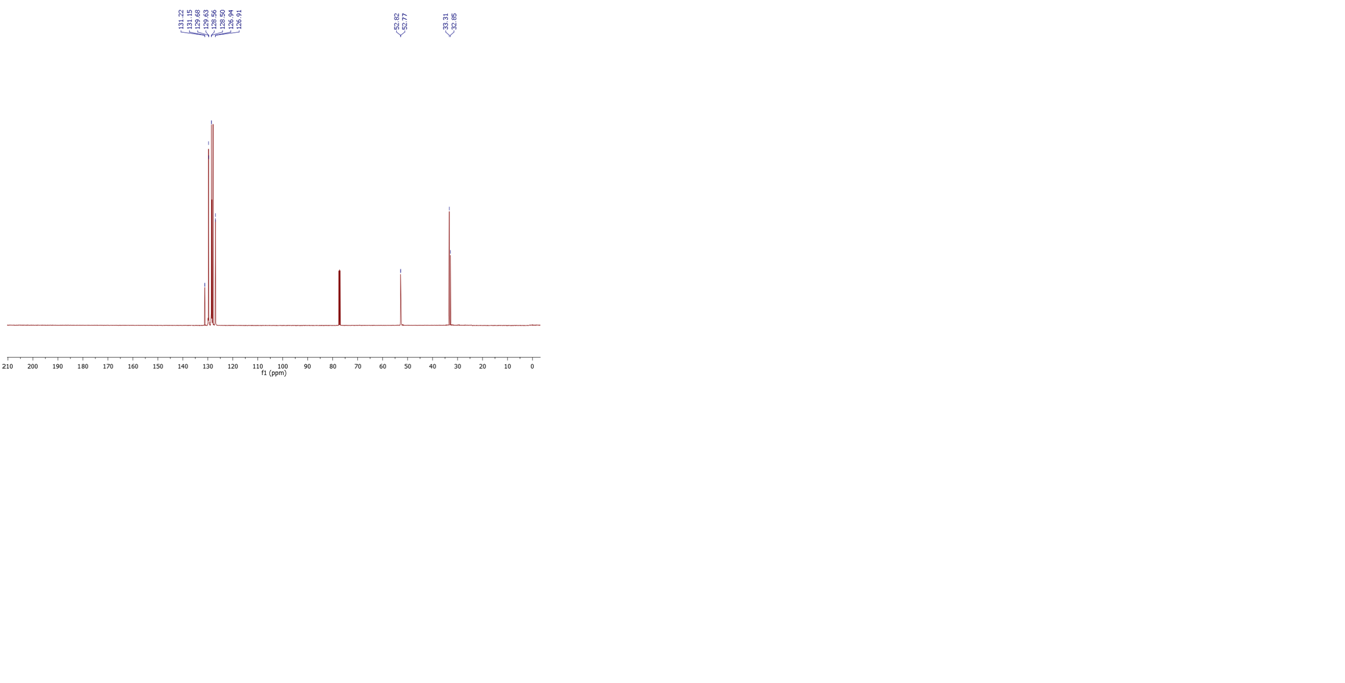
**

**^31^P NMR Spectra of the Dimethyl benzylphosphonate:**

**
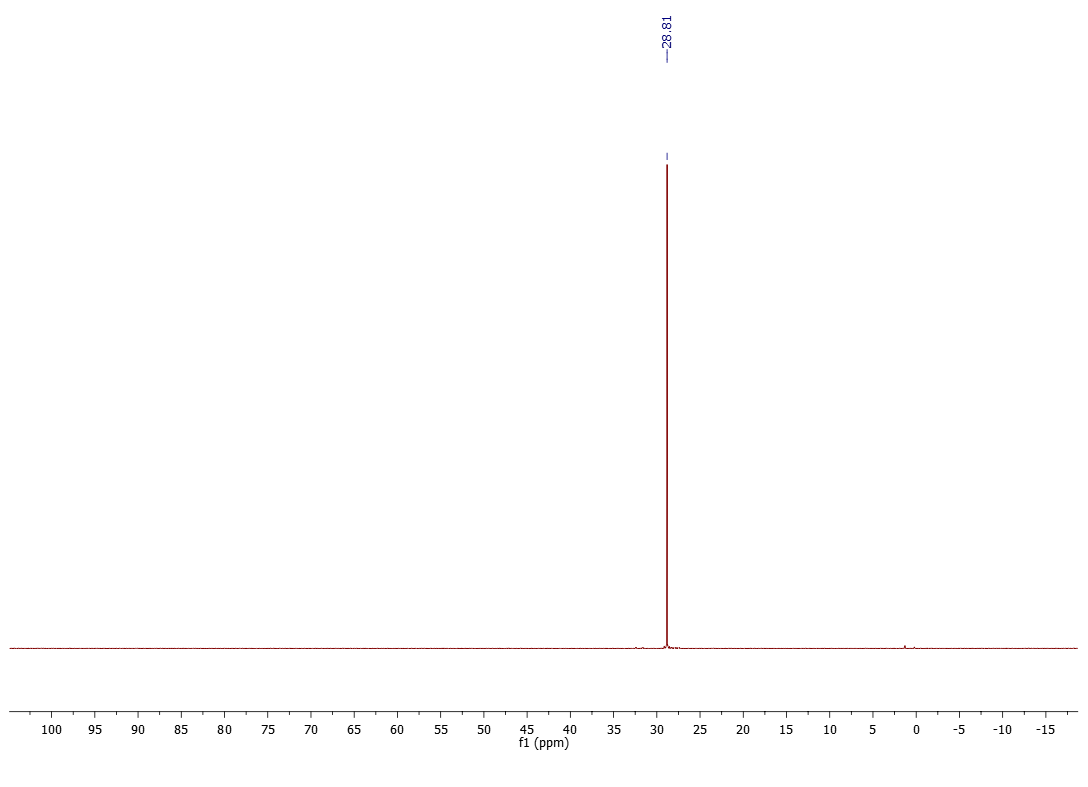
**

**^1^H NMR Spectra of the Dimethyl 4-chlorobenzylphosphonate:**

**
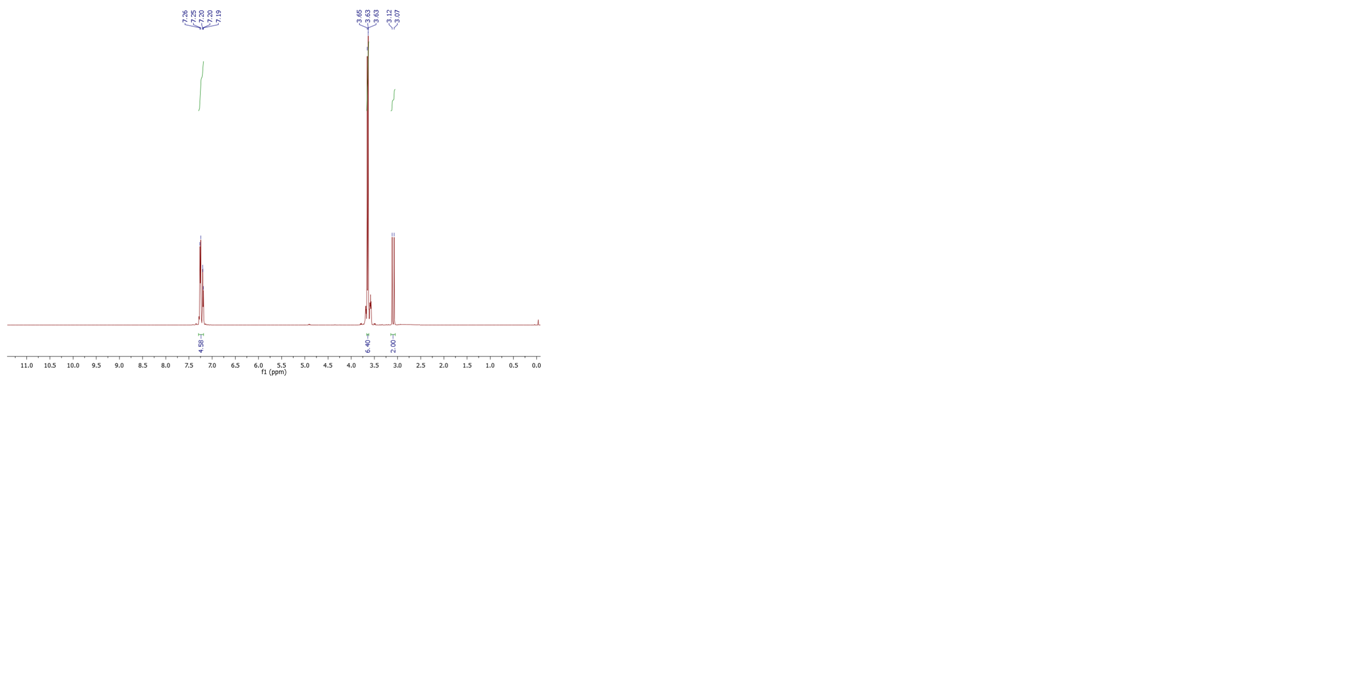
**

**^13^C NMR Spectra of the Dimethyl 4-chlorobenzylphosphonate:**

**
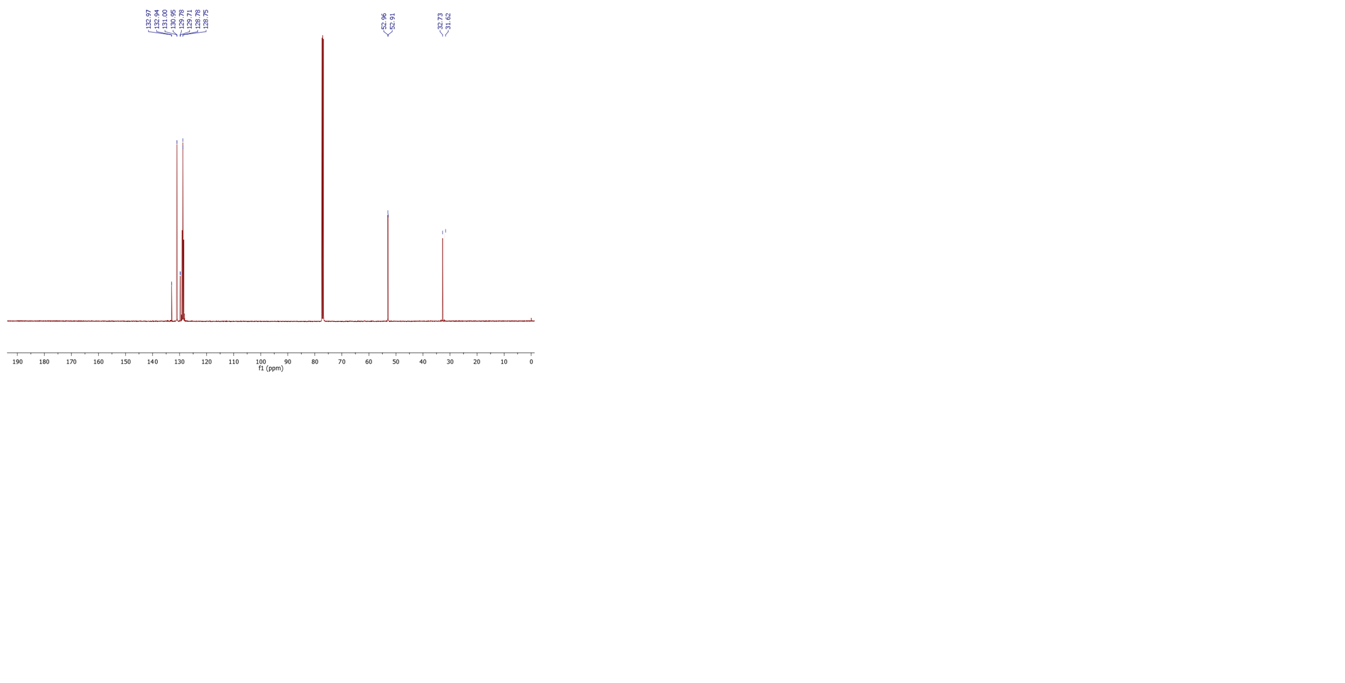
**

**^1^H NMR Spectra of the Dimethyl 3-chlorobenzylphosphonate:**


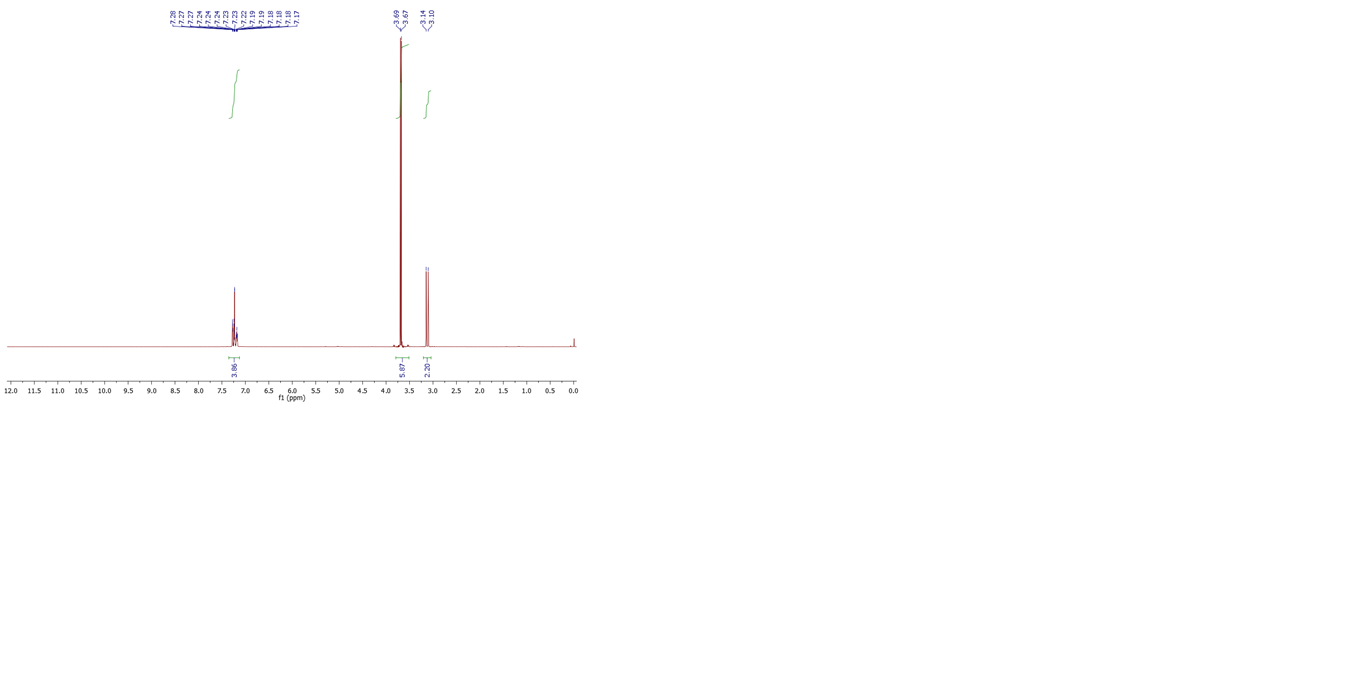


**^13^C NMR Spectra of the Dimethyl 3-chlorobenzylphosphonate:**

**
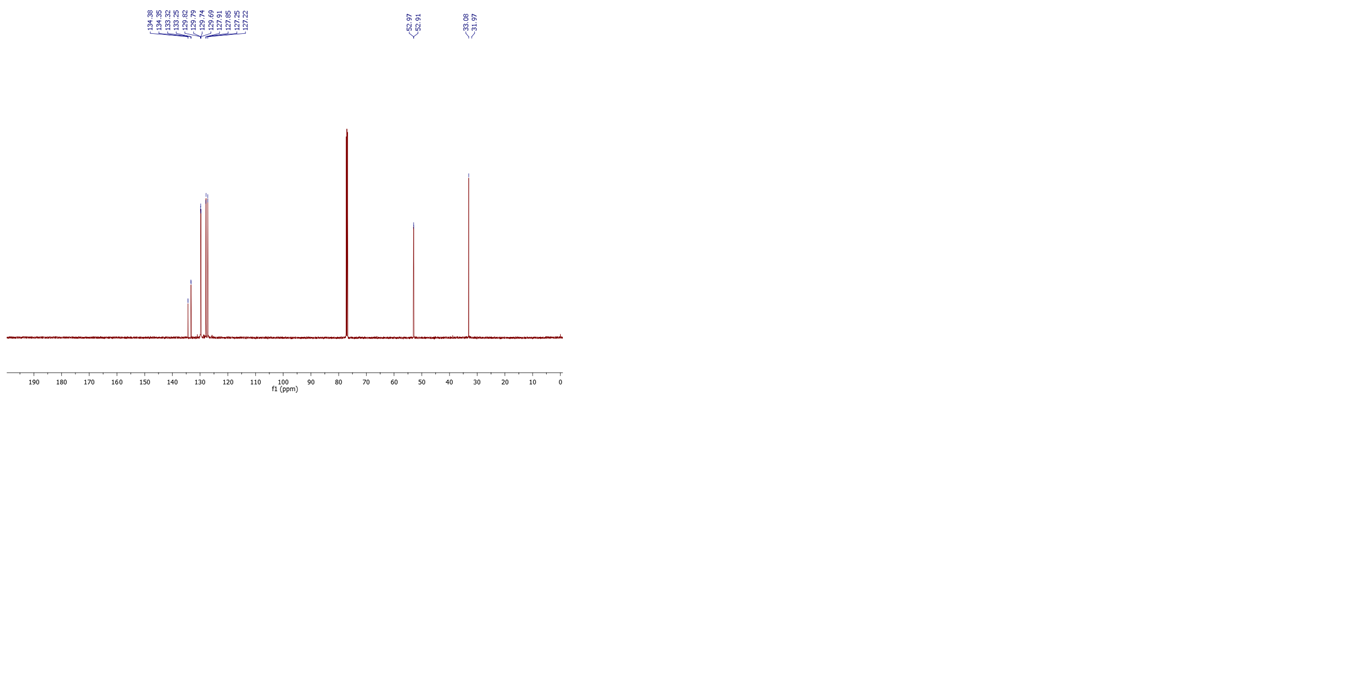
**

**^31^P NMR Spectra of the Dimethyl 3-chlorobenzylphosphonate:**

**
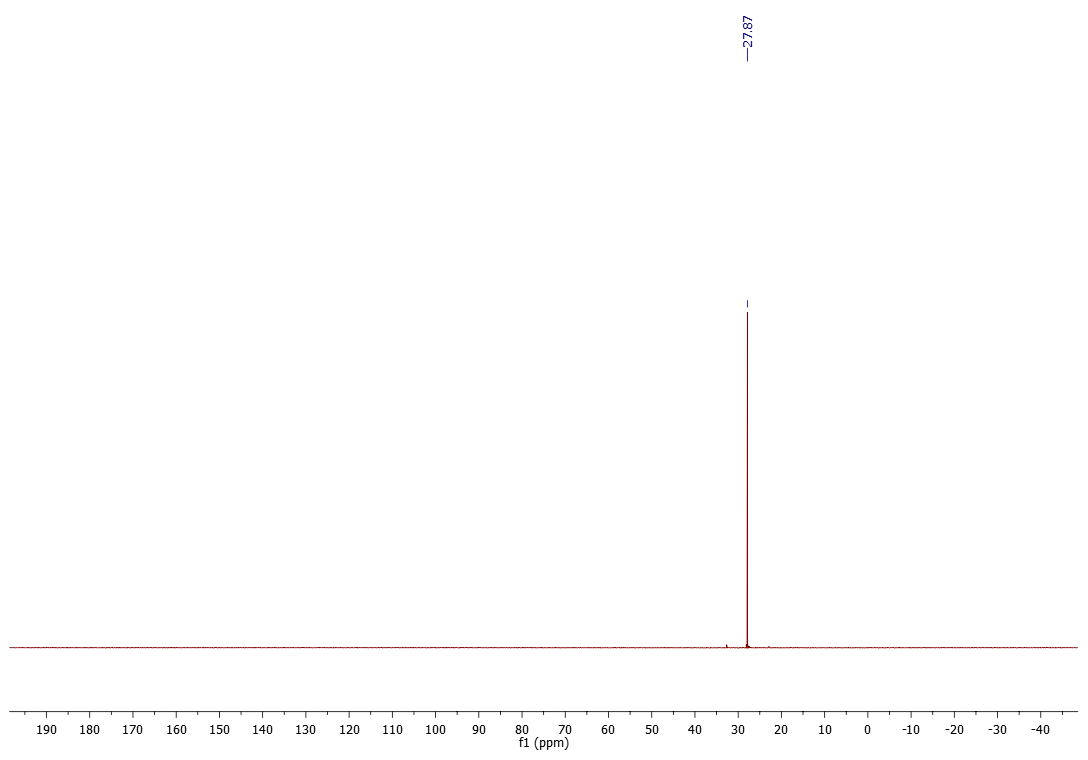
**

**GCMS Spectra of the compounds:**


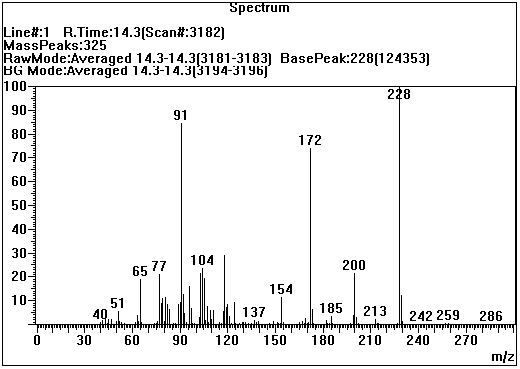


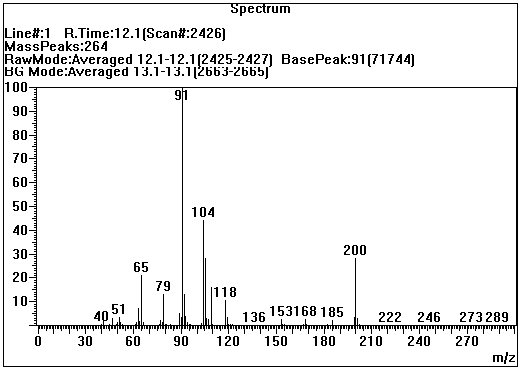


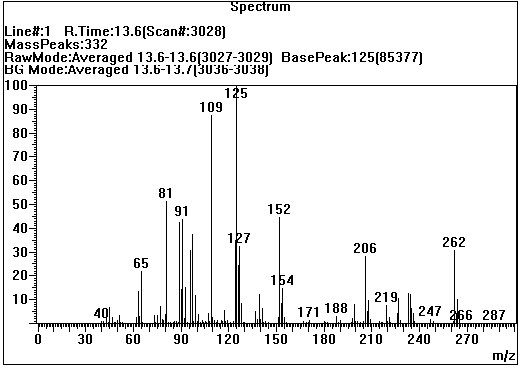


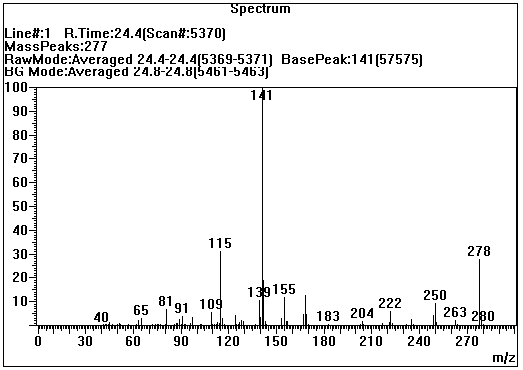


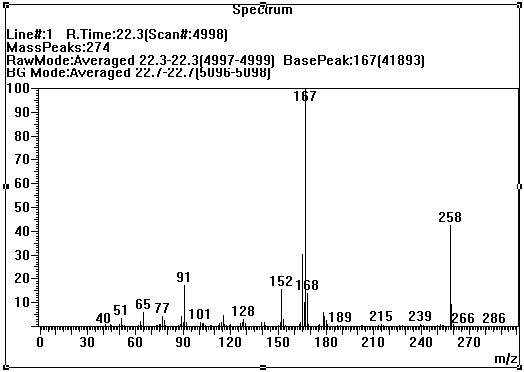


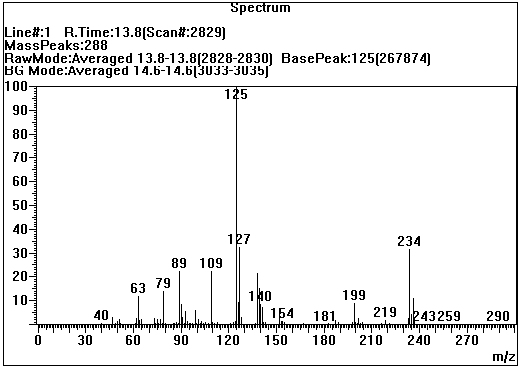


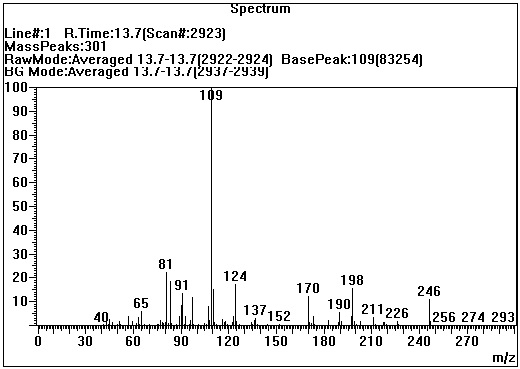


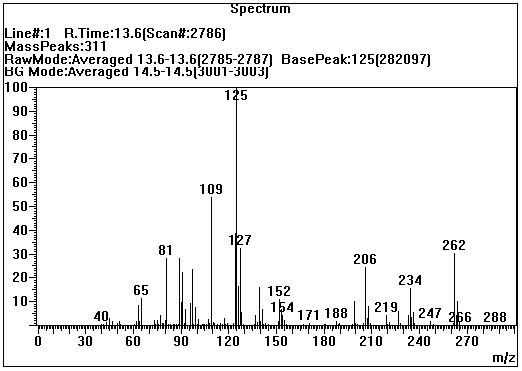


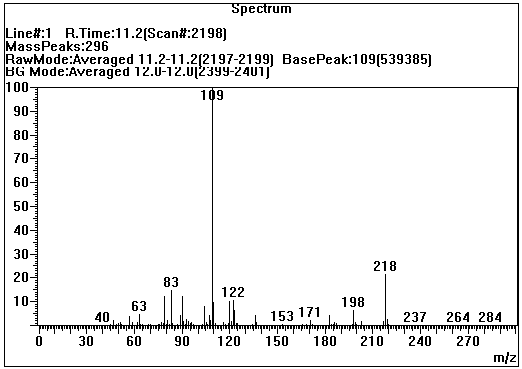


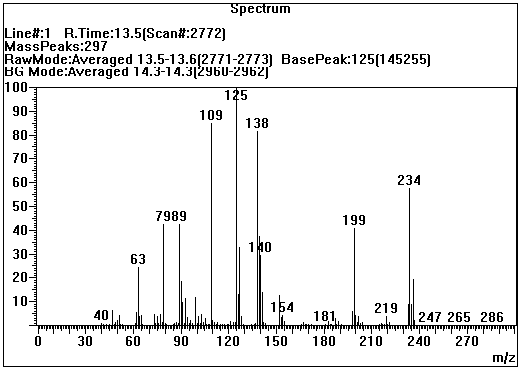

Supplement: Supplementary file 1 [file DataSheet1.DOC]
